# Treatment actions and treatment failure: case studies in the response to severe childhood febrile illness in Mali

**DOI:** 10.1186/1471-2458-12-946

**Published:** 2012-11-05

**Authors:** Amy A Ellis, Sidy Traore, Seydou Doumbia, Sarah L Dalglish, Peter J Winch

**Affiliations:** 1 Social and Behavioral Interventions Program, Department of International Health, Johns Hopkins Bloomberg School of Public Health, Baltimore, MD, USA; 2 World Food Program, Bamako, Mali; 3 Department of Public Health, and Malaria Research and Training Center, Faculty of Medicine, Pharmacy and Dentistry, University of Bamako, Bamako, Mali; 4 Catholic Relief Services, Phnom Penh, Cambodia

**Keywords:** Malaria, Fever, Child survival, Care-seeking behaviors, Treatment pathways, Community health, Mali

## Abstract

**Background:**

Appropriate home management of illness is vital to efforts to control malaria. The strategy of home management relies on caregivers to recognize malaria symptoms, assess severity and promptly seek appropriate care at a health facility if necessary. This paper examines the management of severe febrile illness (presumed malaria) among children under the age of five in rural Koulikoro Region, Mali.

**Methods:**

This research examines in-depth case studies of twenty-five households in which a child recently experienced a severe febrile illness, as well as key informant interviews and focus group discussions with community members. These techniques were used to explore the sequence of treatment steps taken during a severe illness episode and the context in which decisions were made pertaining to pursing treatments and sources of care, while incorporating the perspective and input of the mother as well as the larger household.

**Results:**

Eighty-one participants were recruited in 25 households meeting inclusion criteria. Children's illness episodes involved multiple treatment steps, with an average of 4.4 treatment steps per episode (range: 2–10). For 76% of children, treatment began in the home, but 80% were treated outside the home as a second recourse. Most families used both traditional and modern treatments, administered either inside the home by family members, or by traditional or modern healers. Participants’ stated preference was for modern care, despite high rates of reported treatment failure (52%, n=12), however, traditional treatments were also often deemed appropriate and effective. The most commonly cited barrier to seeking care at health facilities was cost, especially during the rainy season. Financial constraints often led families to use traditional treatments.

**Conclusions:**

Households have few options available to them in moments of overlapping health and economic crises. Public health research and policy should focus on the reducing barriers that inhibit poor households from promptly seeking appropriate health care. Enhancing the quality of care provided at community health facilities and supporting mechanisms by which treatment failures are quickly identified and addressed can contribute to reducing subsequent treatment delays and avoid inappropriate recourse to traditional treatments.

## Background

Malaria is a major cause of child death and disability in Mali. Presumptive malaria accounted for 33.8% (n=10,123) of all recorded consultation diagnoses between 1998 and 2006 in a study in the central Mopti region [1]. In 2009, 68% of reported deaths of Malian children under 5 were attributed to malaria [2]. The effects are particularly severe in Koulikoro and other Sahelian zones, where the rainy season brings six months of high malaria transmission from June to November [3]. A study mapping West Africa’s geographic risk profile for anaemia, to which malaria is a major contributor, identified a large spatial cluster of maximal risk of anaemia (>95%) over an area abutting Koulikoro region, where the risk was nearly as high [4].

Appropriate home management of malaria is a central component of global efforts to control the disease, particularly in settings where access to health facilities is limited. This strategy relies upon caregivers to recognize illness symptoms, assess their severity and take appropriate action, either by initiating early treatment in the home with anti-malarial drugs, or by seeking care at a health facility in the case of severe illness [5,6]. The capacity to properly manage malaria at the household level is dependent upon a multitude of social, economic and other contextual factors. In most settings, symptoms that correspond to uncomplicated malaria are widely recognized as a febrile illness requiring care, which often begins in the home with tepid sponging, herbal remedies, or pharmaceuticals such as anti-malarials, antipyretics and/or analgesics. Many studies have demonstrated this general pattern of recognition and care throughout different regions of Africa, such as in Nigeria [7,8], Ghana [9], Zambia [10], Tanzania [11,12], Ethiopia [13], Kenya [14], and Malawi [15].

Knowledge about malaria recognition, causation and treatment is only one of many factors which contribute to treatment seeking behavior. Even if an illness is identified as malaria, previous illness experience and perceived severity of the ongoing episode affects how the illness is managed [16]. Treatment of malaria in sub-Saharan Africa is strongly related to poverty [17], and cost and accessibility of care are of primary concern and are critical determinants of health care utilization [18]. User fees at health care facilities and the cost of medicines can have a significant impact on patterns of care-seeking for malaria [19,20]. The direct costs of medicines and health services can represent significant liabilities for poorer households, but indirect costs due to travel and lost production may be even more prohibitive [21]. The costs associated with malaria can be particularly dramatic for low-income households, even when little or no charges for public sector primary health care are incurred [22]. Indirect costs due to caretaking can be considerable because of the large number of children who become ill from malaria and who must be cared for by parents or other adults [23]. The ramifications of an episode of malaria may spread well beyond the individual experiencing illness – the household as a whole may be dramatically impacted as members must neglect their usual tasks to care for the ill individual, scramble to compensate for lost production, and reallocate assets to pay for care, food, and treatments [24].

Many other factors can cause already vulnerable households to be even more at risk from the effects of malaria and influence the pursuit of treatment. In Mali, as in many regions of Africa, the rainy season corresponds to the period when food stores and financial resources are scarcest, but the physical demands of the agricultural cycle and the transmission of diseases are at a peak. Sauerborn and colleagues found that despite the higher burden of disease and incidence of malaria during this time, there was a significant drop in heath service utilization and a reduction in both direct and indirect costs incurred per illness episode. Seasonality can also hinder access to health facilities in more obvious ways, such as by hampering transportation and making roads impassable [25].

The treatment of most illnesses begins in the home, and malaria is no exception. Studies examining treatment patterns for malaria indicate that the first response to illness is typically self-treatment. Although the term ‘self-treatment’ is often used in studies, it is not consistently defined, and can indicate anything from home remedies to a self-administered course of anti-malarials or other drugs [26]. Most studies indicate that the first resort to care for uncomplicated malaria is the use of pharmaceuticals in the home, which are often purchased from drug vendors or are left over from previous illness episodes [10,27-30]. In cases of severe illness, traditional healers with the perceived ability to counter spiritual or supernatural illnesses become an important source of care [11,31-35]. Households may attempt to minimize the impact of illness costs by attempting to compensate for lost labor or by selling assets, such as livestock [36]. Material assets are often not the only resources drawn upon to cope with costs associated with illness. Social resources in the form of relationships or networks may be accessed in order to borrow assets or incur debt, and potential or future income may be sacrificed to mobilize immediate resources [37].

In rural areas of Mali, there are almost no formal sector private providers such as private clinics, private pharmacies or mission hospitals. Government health facilities and community health workers are essentially the only formal sector health care providers. There are referral hospitals at the regional and district levels. At the sub-district level, simple community health centers (Centre de Santé Communautaire or CSCom) are typically staffed by a head nurse, an auxiliary nurse-midwife, and a nurse’s aide. Physicians are generally only found in district and regional referral hospitals. The informal sector is an important source of medical care, and includes market stalls, shops and ambulatory vendors. A wide variety of modern medicines, including anti-malarials, are available in the informal sector - a large portion of which may be counterfeit, expired or spoiled. Traditional healers are also a frequent source of care, and include herbalists and ‘old wise women’, as well as healers that deal with illnesses of perceived supernatural causes. Many people have knowledge of common traditional treatments, which are typically teas consisting of boiled leaves, herbs and bark.

This research examines the management of twenty-five episodes of severe febrile illness (presumed malaria) in children in Koulikoro Region, Mali, and explores the sequence of treatment steps taken during each illness episode. We report patterns in care-seeking and participants’ perceptions of care-seeking options, both inside and outside the home, using traditional and modern remedies. Barriers to formal care-seeking, and notably the cost of care, are discussed.

## Methods

An in-depth qualitative study was conducted in the Koulikoro Region of Mali from October 2006 through January 2007, coinciding with the end of the rainy season and a peak in the number of severe malaria cases. Twenty-five households, located within four villages, participated in the study. Villages ranged in size from 870 to 4,600 inhabitants, and household livelihoods were based primarily on subsistence farming of millet and seasonal gardening. Two villages had a functioning public health facility within the village, and two villages were located 5 kilometers away from their respective local health center.

Purposive sampling was used to select households in which one child under the age of five had experienced a severe febrile illness within the past two weeks. Households were identified with the assistance of health agents, traditional healers, village leaders and community members so as to include a variety of households seeking treatment from different sources of care.

An illness was labeled as a ‘severe febrile illness’ if it met the Integrated Management of Childhood Illness (IMCI) classification of severe malaria. Because laboratory diagnosis was not possible, a severe febrile illness was treated as a proxy for a case of severe malaria [38]. In addition to meeting the IMCI classification of severe malaria, most cases were also identified as malaria at a health facility. However, it is possible that some cases were due to causes other than, or in addition to, malaria, such as pneumonia or meningitis.

In households meeting the inclusion criteria, two interviews were conducted with the mother of the child. One interview was conducted with the father, as well as all household members who were identified as having been involved in the illness episode at any point (106 interviews with 81 informants). Beyond the child’s parents, household members involved in the illness episode were primarily grandparents, uncles, aunts, and co-wives. This approach was taken because the majority of research and programming related to child health focuses specifically on the primary caretaker of the child, usually the mother. However, the study sought to generate a broader, more holistic perspective of how households manage severe childhood illness by including anyone who had been involved in some way during the illness episode. Informants were asked to describe illness symptoms, treatment steps, decision making processes and the participation of different household members so as to create one comprehensive narrative and timeline of the illness episode. Additionally, key-informant interviews (n=9) were carried out with heath agents, village leaders and traditional healers, and focus group discussions (n=8) were conducted with male household heads, grandmothers, fathers and mothers.

All data collection activities were conducted individually and recorded in the local language, Bambara, and were later translated and transcribed verbatim into French. Interviews and focus groups were content analyzed with the use of Atlas.ti [39]. Ethical approval for the study was granted by the Johns Hopkins University Bloomberg School of Public Health Committee on Human Research and the University of Bamako Faculty of Medicine, Pharmacy, and Dentistry Internal Review Board. Verbal consent was obtained from local health officials, village leaders, and individual participants.

## Results

Characteristics of households and participants are displayed in Table 1. Twenty-five household case studies were included, encompassing interviews with 25 mothers and 56 other household members (N=81), with an average of 3.2 participants per household (range: 1 – 7). Mothers tended to be younger than other participants, with an average age of 30.7 compared to 47.4 for other participants. Most households’ primary source of income was from farming (84%); household organization was split between monogamous (56%) and polygamous (44%) marriages. Just over half of households (52%) were located in a village with a health center.

**Table 1:** Demographic Characteristics of Participants (n=81) and Households (n=25)

**Participant characteristics (n=81)**	***n***	***Average Age (Range)***
Age (years)		
Mothers	25 (30.9%)	30.7 (19–40)
Other participants	56 (69.1%)	47.4 (24–70)
Child	--	2.3 (0.5– 5)
Participants per household	--	3.2 (1 – 7)
**Household characteristics (n=25)**	***n***	***Percent***
Primary source of household income		
Farming	21	84%
Teacher	2	8%
Market vendor	1	4%
Motorcycle repair	1	4%
Family structure		
Monogamous	14	56%
Polygamous	11	44%
Health centre in household’s village	13	52%

**Figure 1:**
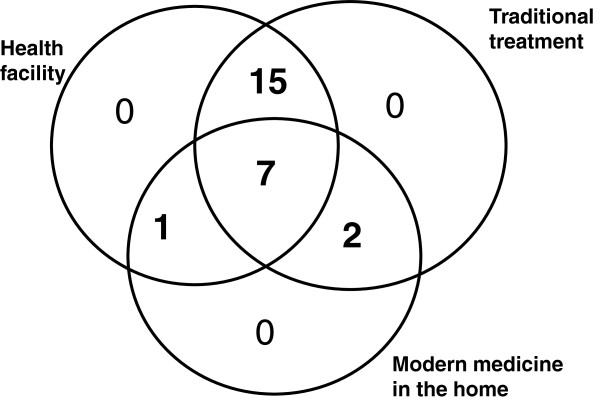
Overlap of traditional and modern medicines in treating severe febrile illness in children under 5 in Koulikoro, Mali (n=25).

The duration of the children’s illness episodes varied significantly. Eleven of 25 illness episodes (44%) persisted for longer than one month; three children died. Twenty-four of the twenty-five children were treated at a health facility at some point during their illness. Of these, 23 children were also treated using traditional treatments. The remaining child was treated only at home using both traditional treatments and modern pharmaceuticals (see Figure 1). In 76% of illness episodes (n=19), the first treatment step took place inside the home, using either traditional treatments (48%, n=12) or modern pharmaceuticals (28%, n=7).

### Treatment pathways

**Figure 2:**
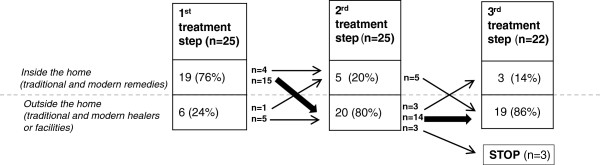
Treatment pathways for severe febrile illness in children in Koulikoro, Mali.

Children’s illness episodes involved multiple treatment steps, with an average of 4.4 treatment steps per episode (range: 2–10). Treatment steps were classified into two categories: 1) home treatment, either with traditional treatments or modern medicines, and 2) treatment outside the home, either by a traditional healer or at a health center (local, regional, referral, etc.) Figure 2 applies these categories to children’s first three treatment steps (n=25), to demonstrate aggregate pathways of treatment-seeking behavior. For most children (76%), treatment began in the home; however, 80% of second treatment steps took place outside the home, half in formal facilities and half using traditional healers. By the third step, an even smaller proportion (14%, n=3) of treatments were home-based, demonstrating an escalating reliance on external sources of care as the perceived severity of the illness episode increased.

Treatment steps typically included recourse to a variety of different sources of care, and households rarely limited themselves to either modern or traditional therapies. Overlap between different sources of care is demonstrated in Figure 1. Ten children (40%) received any type of modern medicine in the home (usually chloroquine and/or acetaminophen) and 24 children (96%) were treated using traditional treatments. Additionally, twenty-three children (92%) received care at a health facility, indicating the overlapping use of these three sources of care. While special attention was paid to identifying the sequence in which each treatment step was initiated, in many cases, treatments overlapped. For example, often the first treatment step of traditional treatment administered in the home was not discontinued when treatment with modern medicine commenced.

Of the twenty-three children who were treated at a health facility, twelve (52%) reportedly experienced subsequent treatment failure and eight later returned to the same or another health facility, which also did not always result in a final cure. Children brought to the health center earlier in their illness appeared to fare better than those brought later; however, this relatively low cure rate for health facilities likely explains some participants’ mistrust in formal health care, discussed below.

**Figure 3:**
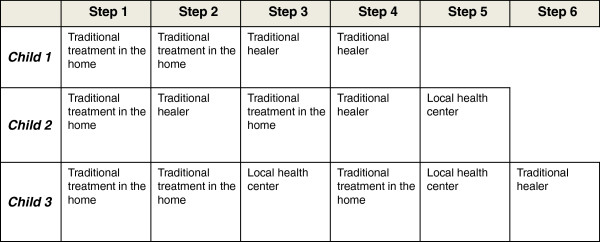
Treatment steps for children deceased from their illness (n=3).

Of the children experiencing reported treatment failures, three died of their illness. Although none died on site at the health facility, two children were treated and discharged after it was determined that nothing else could be done, and died within 24 hours. One child was not brought to a health center at any point during the illness episode because of financial barriers. The treatment steps of these three children are displayed in Figure 3, and suggest a reliance on traditional treatment and delays in bringing the child to a health facility, compared to the rest of the sample children.

### Signs of severity and convulsions

Many illnesses were managed outside of the formal health sector for long periods of time, either with traditional treatments or modern pharmaceuticals. Perceived severity of the illness was the most influential factor instigating treatment seeking at the health center, according to interviews. Signs of severity deemed alarming included excessive vomiting, extreme fever, inability to eat, persistent symptoms without improvement, and, notably, convulsions.

Convulsions were universally regarded as a sign of extreme severity, and the presence of this symptom always resulted in urgent care-seeking – either from a health facility or a traditional healer. If convulsions occurred during the illness episode, caregivers often (but not always) responded by seeking care at a health facility. However, if the child relapsed and convulsions occurred again after care had been sought at the health center, parents were less likely to return there, believing that health facility medicines were ineffective or that the illness has been misdiagnosed and therefore treated incorrectly. In illness episodes with no convulsions, caregivers often mentioned previous experience with convulsions as playing a role in their decision to seek facility-based care before the illness progressed to such a severe state.

### Treatment failure in the formal health sector

Preference for modern treatment at a health facility was frequently stated, and 23 of 25 families brought their child to health center at some point during the illness episode. Compared to traditional treatment, modern medicines were often considered “faster acting” and “less tiring.” One father who expressed a strong preference for modern medicine stated:

"In regards to treatment, if I have the money, I go to the health center because I prefer this to the traditional healer. If I accept going to the traditional healer, it is because I do not have money. Running here, running there to treat with traditional remedies, all of this is because of a lack of means. Otherwise, if one has the means, you do not discuss – you execute. (Father, 14M1)"

However, treatment at health facilities was not always successful: 52% of children (n=12) treated in a health facility reportedly experienced treatment failure. Informants accounted for this in a variety of ways, citing the increased virulence of certain diseases, innate differences in people that cause them to respond differently to treatments, and misdiagnosis of the illness. Regardless of the reason, treatment failure often diminished faith in modern medicine and discouraged future care-seeking there. Two informants described their disappointment after a visit to the community health center failed to cure the illness of a sick child in their household:

"Often, I am not satisfied at all; this is why I say that I am discouraged. When you go to them for a headache, the health agents will often give you medicines for a stomachache. Can this cure this illness? Often they give injections that do not cure the illness. They try to treat you by trial and error. (Uncle, 05M3)"

"For this nyama of my daughter, we always go to the health center, but the child is not cured completely. This is what causes me to not believe the health agents any more. (Mother, 20A2)"

Treatment failures in the formal health sector often led informants to conclude that traditional treatments would have been a better choice, or that the child’s disease (particularly *nyama*, whose symptoms are believed to overlap with those of severe malaria) could not be treated using modern care. One grandmother explained:

"For three days, the medicines from the health center were given to the child, but they did not work. The health workers said that this illness was sumaya [malaria]. But no, ohh, it was nyama – if it’s nyama, the medicines that were given there cannot be effective. Oh, for nyama, you must give traditional therapy because the health workers do not know nyama. (Grandmother, 11M1)"

Others doubted the ability of health centers to cure all patients:

"You know, treatment is the sort of thing where there are treatments that work well for certain people, while the same treatment worsens the illness of another. The blood of one person can support one treatment, but the blood of other is not able to tolerate the treatment. Each person has their own blood – it is this that complicates the illness. (Mother, 06A1)"

Interestingly, even informants who downplayed the efficacy of modern medicines for some illnesses still professed to buy them:

"All of our children’s illnesses are managed the same way; the vendors who come to our village here selling candies and cigarettes also sell medicines. We buy some tablets from these vendors for illnesses that are a little serious but that don’t require going to the health center. But often we say that these modern medicines are of no use, we say that it does not treat our illnesses. When you use these medicines against certain illnesses, they do nothing. (Mother, 05A2)"

### Economic barriers’ effect on treatment choices

Eighty-four percent (n=66) of informants stated that the foremost obstacle to seeking care at a health center was financial (either not having money or care was too expensive). The cost of treatment for each child varied dramatically (range: <1$ - 150$), however, most health care expenditures ranged between $4-7. Home treatment with modern medicines, as well as treatment from traditional healers, rarely exceeded $1 per treatment step. Financial implications of the illness episode were frequently the source of conflict in the household. One father, who argued with his brother over treatment for his sick child, stated: “If there are diverging ideas about where to treat a child - this is always about the problem of money.”

Seasonality was an important factor in financial decisions about care: many informants explained that “not all moments are the same.” If an illness occurs at a time when resources are available, care may be sought directly at a health center with little hesitation or discussion. However, if an illness coincides during a time of scarcity, for example during the rainy season, financial barriers may delay or prohibit prompt care-seeking:

"This illness began during the rainy season, and there was nothing. Before the harvest, we Bambara earn nothing and any money there is must go to pay for food …. The illness found us when there was truly an economic crisis. (Mother, 01A2)"

In the absence of household resources, informants collectively stated that they can seek credit among friends and family, or sell household goods in order to pursue treatment, and several households did borrow money in order to bring their child to the health center.

Many informants said the cost of treatment at the health center forced them to turn to traditional care. One grandfather explained, “Our people do the treatment that costs less; the health center is expensive… If you do not have the means, you must content yourself with traditional therapy.”

Furthermore, whereas sick children would be turned away from a health facility, participants said traditional healers could be relied upon to provide treatment “with a sense of pity, fraternity and good neighborliness.” Conversely, formal health structures were sometimes resented for their lack of empathy:

"Regardless of the severity of the illness, if you do not pay the money, the health workers will not treat your sickness. If you do not have the means, your sick child is going to die beneath the eyes of the doctor. He is not going to cure him because you did not pay the cost. This does not exist among us Bambara, where the traditional healer will treat your sick child without money and you can reimburse him afterwards. (Grandfather, 2CD)"

## Discussion

A study of health seeking behavior by Ryan in rural Cameroon illustrates several characteristics of the management of illness at the household level in medically pluralistic, resource constrained settings similar to Mali. First, people delay treatment action in order to better identify the illness and categorize it into a certain treatment type. Caregivers may wait until the absolute last moment to seek care at a health center in hopes that less expensive home treatments will prove sufficient. Second, people attempt to minimize costs by choosing treatments that are less expensive or by reducing the number of treatments. Unfortunately, by delaying recourse to effective care at a health center early by suppressing, but not curing, the illness with use of traditional treatments or sub-therapeutic or ineffective doses of pharmaceuticals, an uncomplicated illness frequently progresses to a severe, life-threatening illness that requires more expensive treatment to cure. Thirdly, people maximize treatment variety in the hopes of finding at least one treatment that helps cure the illness [40].

All of these phenomena were also observed in this research. In these case studies, families may seek care at a health center if initial efforts in the home do not alleviate symptoms, but may again return to traditional therapies if treatment received at the health center is perceived to have failed. Treatment steps vacillate between various sources of care, and traditional and modern therapies may be applied simultaneously at different points throughout the episode. This is in line with other findings in West Africa, for example in a study in Nigeria, where the preferred sources for malaria treatment services were in public hospitals (30.5%) and primary healthcare centers (18.1%). Traditional healers (4.8%) and patent medicine dealers (4.2%) were the least preferred strategies for improving malaria treatment [41].

In this study, despite a preference for seeking care at a health center, where treatment was perceived as being faster acting and more effective, direct and indirect costs associated with care-seeking there frequently compelled households to address symptoms with treatments accessible outside of the formal health sector. This strategy allowed households to postpone recourse to expensive treatment at a health center, however, often to the point that the illness progressed to a life-threatening episode. In Burkina Faso, financial constraints were observed as being particularly problematic during the rainy season, when health problems are common but available resources are at their most limited [36,42]. The severe and sudden manifestation of acute illnesses leaves poor households little time to generate money, liquidate assets, or find alternate sources of labor, which can elevate a severe illness from being a manageable problem to a true household crisis. Rather than being due to a lack of knowledge, the ‘denial’ of disease and the sub-optimal treatment of illness is often the result of household strategies to cope with illness in the presence of resource constraints and inaccessible health care [43]. And though households may turn to neighbors for assistance in times of difficulty, such social resources may offer little relief at a time when entire communities are in a fragile state.

Reported treatment failure in formal health care settings, as occurred in 52% (n=12) of cases in this study, is common in other contexts as well. In rural Kenya, 58% of children who succumbed to fatal malaria were treated by a trained health provider [44]. Similarly high rates of care-seeking were observed in rural Tanzania, where more than three-quarters of children who died received treatment at a health facility [45,46]. When modern treatments fail, the diagnosis is often reevaluated, or traditional treatments are perceived as being more appropriate for the ill child, motivating care-seeking outside of the formal health sector [33,47]. Although some explanations cast the blame away from the efficacy and quality of health services themselves, resentment, distrust and reduced confidence in community health centers inevitably emerges after treatment failures.

### Study limitations

This study’s qualitative methodology implies a smaller sample size than found in most quantitative studies, although the inclusion of 81 participants who discussed the cases of 25 severely sick children is not small by qualitative standards. Cases were purposively sampled and are not thus necessarily representative of severe childhood illness in Mali. Although the research was conducted retrospectively, it was designed to minimize recall bias by initiating case studies within two weeks of the child’s last symptoms; some case studies were initiated while the illness was still ongoing.

It was occasionally difficult to identify each discrete treatment step – particularly for treatment sought outside the formal health sector and for illnesses of a long duration. However, participants from the same household were able to complete or complement one another’s account, providing a more or less complete retelling of the episode. As household members were sometimes more involved in one aspect of the illness episode than another, illness narratives often included varying perspectives on the same episode.

Some children’s diagnoses of malaria were not laboratory-confirmed, and it is possible that their illnesses were due to another pathogen in sequential or concurrent infections. Indeed, some parents described their child’s illness as recurrent or even congenital. Lastly, many of the reported treatment failures could be due to the fact that artemisinin-combination therapies (ACTs) were not yet present in most rural health facilities. These more effective medicines are now standard treatment in Malian public health facilities, so there are likely fewer treatment failures for malaria as of present.

## Conclusion

This study provides evidence of high rates of treatment-seeking from health facilities in the case of severe febrile illness in children in rural Mali – as well as treatment failure in these same facilities. The high prevalence of reported treatment failure may justify participants’ expressions of skepticism as to the efficacy of modern medicine, although this remained by far the preferred treatment. Care-seeking pathways tended to begin in the home; later treatment steps were more likely to take place outside the home. In most cases, caregivers used both traditional and modern remedies, often simultaneously. In illness episodes that were prolonged, delays in care-seeking were most often attributed to financial barriers. Direct and indirect cost barriers are highest during the rainy season, a difficult period for many rural households, as food and financial reserves run low but the harvest is not yet available. Caregivers attempted to minimize unnecessary costs by repeatedly trying less expensive treatments in the home before seeking costly care at the health center.

While the availability of ACTs in Mali and an accompanying decrease in user fees should contribute to reducing delays in care-seeking and treatment failure, larger systemic issues relating to the ability of public health facilities to properly diagnose, manage and treat disease will persist. In poor settings such as rural Mali, decreasing user fees will reduce some, but not all of the direct and indirect costs associated with care-seeking. Furthermore, concerns about the quality of care provided and the ability of lower level health facilities to respond to treatment failures when they do occur will also significantly influence care-seeking behaviors.

Strategies for encouraging prompt health seeking, as well as making care more accessible to households, especially in times of hardship and reduced resources, must continue to be a priority. However, getting households to bring sick children to the health center is only the first step. Ensuring that children receive necessary follow up care after they leave a health facility, and the best approaches for responding to and managing cases of treatment failure, must also be considered. This study describes household care-seeking patterns for childhood illness in Mali that have been identified in other settings, indicating that although health systems may differ or change, trends in treatment-seeking pathways will likely persist. This underlines the importance of understanding community and health system factors not separately but together, so as to create a comprehensive strategy to reduce child mortality and morbidity in rural settings.

## Competing interests

The authors declare they have no competing interests.

## Authors’ contributions

AE and PW developed the detailed design of this study, and the final research instruments. SD provided input and actively commented on design details and this paper. ST provided input into the design of the study and data collection. AE lead the overall study, including data collection, overseeing training of field researchers, analysis and reporting. SLD performed some analysis and contributed to reporting. All authors read and approved the final manuscript except ST, who is recently deceased.

## Pre-publication history

The pre-publication history for this paper can be accessed here:

http://www.biomedcentral.com/1471-2458/12/946/prepub
